# Help others—be happy? The effect of altruistic behavior on happiness across cultures

**DOI:** 10.3389/fpsyg.2023.1156661

**Published:** 2023-06-23

**Authors:** Merav Weiss-Sidi, Hila Riemer

**Affiliations:** Department of Management, Ben-Gurion University of the Negev, Beersheba, Israel

**Keywords:** help, altruism, happiness, culture, individualism-collectivism

## Abstract

Research has established that altruistic behavior increases happiness. We examined this phenomenon across cultures, differentiating between individualistic and collectivist cultures. We propose that cultural variations in the notion of altruism lead to different effects of helping on the helper’s happiness. For individualists, altruism is linked to self-interest (“impure” altruism), and helping others results in increased happiness for the helper. For collectivists, altruism is focused on the recipient (“pure” altruism), and helping others is less likely to enhance the helper’s happiness. Four studies support our predictions. Study 1 measured the dispositions toward altruism among people with various cultural orientations. Consistent with our predictions, the findings showed that individualism (collectivism) was positively associated with tendencies reflecting more “impure” (“pure”) altruism. Two experimental studies then examined the moderating role of cultural orientation on the effect of spending money on oneself versus others (Study 2) or of doing a kind action (making tea for oneself versus others; Study 3). Both experimental studies demonstrated that altruistic behavior had a positive effect on happiness for individualists but not for collectivists. Finally, Study 4, which utilized data from the World Values Survey to examine the altruism–happiness link in various countries, displayed a stronger link between altruistic behavior and happiness in individualistic (vs. collectivist) cultures. Altogether, this research sheds light on cultural differences in the display of altruism, revealing different motivations for and consequences of altruistic behaviors.

## Introduction

Consider the following quotes:

*“Since you get more joy out of giving joy to others, you should put a good deal of thought into the happiness that you are able to give.” Eleanor Roosevelt*

*“Our prime purpose in this life is to help others.” The Dalai Lama*

These two quotes describe distinct perspectives on altruistic behavior. The first quote focuses on the self-benefit for the helper (the giver) and emphasizes that the prime motivation for helping others is the joy the helper would gain. The second quote, by contrast, presents helping others as the prime motivation in people’s lives, regardless of their self-benefit. These two contrasting perspectives presented by the Westerner Eleanor Roosevelt and the Easterner the Dalai Lama reflect cultural differences in the motivation for and the consequences of such behavior. In the current research, we argue that because the notion of altruism differs across cultures, the outcome of helping behavior—particularly its effect on happiness—vary across cultures.

Research has shown that altruistic behavior increases the helper’s happiness and promotes positive emotions (for a review, see Aknin and Whillans, 2020). This effect has been demonstrated in a variety of altruistic behaviors, including volunteering (Huang, 2018), donating blood (Buyx, 2009), giving to charity (Liu and Aaker, 2008), spending money on others (Dunn et al., 2008), and making small gestures, such as offering coffee, being kind, or making someone smile (Rudd et al., 2014). Studies have examined the altruism–happiness link in various social groups. Aknin et al. (2013a) found an association in 120 out of 136 countries and concluded that this link does not depend on a country’s wealth. Another study (Aknin et al., 2015) showed that the association between altruism and happiness, as demonstrated among Canadians, was also observed in a rural area on Tanna Island in Vanuatu (a small nation in the South Pacific). This evidence has led researchers to suggest a “possible psychological universal” (Aknin et al., 2013a, p. 646). However, acts of helping involve the interactions of people within a social environment, and is therefore related to personal and societal aspects, such as motivations, values, norms, self-view, and emotions—all of which are culture-dependent (Markus and Kitayama, 1991; Schwartz, 1992; Triandis, 1995). This calls for further consideration of cultural differences in the direction or degree of the relationships between altruism–happiness.

A common classification of cultures relies on the individualism–collectivism dimension, which refers to the degree of integration among the members of a social group (Hofstede, 2001). In individualistic cultures (prevalent in Western countries), people are autonomous, “free” entities who are focused on themselves and on fulfilling their personal goals. Collectivist cultures (prevalent in Eastern countries), on the other hand, feature strong and harmonious connections among group members. Therefore, people in collectivist cultures emphasize the relationships within their social groups and tend to subordinate personal goals to the goals and needs of others (Markus and Kitayama, 1991; Triandis, 1995; Hofstede, 2001). These fundamental cultural differences are evident in broad psychological processes, including information processing (Zajenkowska et al., 2021), attitude formation (Kim and Yim, 2022), motivation (Wiwad and Aknin, 2017), and emotions (see Mesquita, 2022), all of which ultimately influence behavior.

We propose and show that the cultural differences between individualists and collectivists manifest in the notion of altruism. Specifically, we suggest that collectivists display a more “pure” form of altruism—a term introduced by Sisson (1910) to reflect that such altruism emphasizes the benefit to others; by contrast, individualists display a more “impure” form of altruism (Andreoni, 1989, 1990; Asproudis, 2011), reflecting a greater focus on benefiting the helper (see also Hartmann et al., 2017). These distinct notions of altruism convey different focal motivations for altruist behaviors. That is, because collectivists’ essence is to be embedded with and attuned to others, their altruistic behavior is focused on benefiting others (rather than themselves). Collectivists thus engage in altruistic behavior frequently (e.g., Espinosa et al., 2022) and are therefore practiced in doing so. Collectivists’ embeddedness with others and their practicing of altruistic behavior lead them to engage in altruistic behavior in a more intuitive unintended way (Riemer and Shavitt, 2011; Riemer et al., 2014). Consequently, collectivists’ altruistic behavior is less salient, and they tend not to devote a great deal of attention to their altruistic behaviors. This, in turn, limits the impact of these behaviors on their internal state in general, and particularly on their level of happiness (Taylor and Fiske, 1978). We, therefore, argue that there is a relatively low likelihood that collectivists’ altruistic behavior will result in an increase in the helper’s own happiness. By contrast, because individualists’ essence is to be separated and unique from others, and to enhance the self (rather than others), their altruistic behavior is focused on benefiting themselves (rather than others). Individualists thus engage in altruistic behavior less frequently than collectivists, making such behavior more unusual (e.g., Espinosa et al., 2022). Individualists’ altruistic behavior, therefore, tends to be more salient and to attract more attention, which in turn enhances the impact on the helper’s internal state (Taylor and Fiske, 1978). Moreover, views of happiness differ across cultures, such that individualists (vs. collectivists) ascribe greater importance to their own happiness and tend to seek opportunities to enhance their happiness (Oishi and Diener, 2001; Delle Fave et al., 2016). Consequently, there is a relatively high likelihood that individualist altruistic behavior will enhance their own happiness. Thus, we argue that the effect of altruistic behavior on the helper’s happiness will be smaller among collectivists than among individualists.

Next, we review the literature that forms the basis for our conceptualization and describe four studies supporting our propositions. The first study provides evidence of cultural differences relating to the notion of altruism. The other three studies use various methodologies to demonstrate the moderating role of culture in the effect of altruistic behavior on happiness.

## Theoretical background and hypotheses development

### The concept of altruism across cultures

Altruism involves acting in a manner that will benefit others or increase another person’s wellbeing and welfare (Batson and Shaw, 1991). Although altruistic behavior aims to benefit another person, its motivation may come from different sources. Indeed, research has established that behaviors that appear genuinely altruistic may be driven by self-focused motives (Batson and Shaw, 1991), such as monetary benefits (Gneezy et al., 2011), reputation (Griskevicius et al., 2010), reduced concern of social sanction (Bénabou and Tirole, 2006), and positive emotions (Batson, 1987; De Groot and Steg, 2009).

There are two broad types of altruistic motivation. The first is driven by empathic and selfless concern for others; it focuses on enhancing a benefit to the recipient, while any self-benefit is unintended. This type of altruism is described as “pure” altruism (Sisson, 1910; Cialdini et al., 1987; Batson and Shaw, 1991; Batson et al., 2015; Natter and Kaufmann, 2015; Ottoni-Wilhelm et al., 2017). The second type of altruism is self-focused; its ultimate goal is to benefit the self, while benefiting others is purely instrumental. This type of altruism has been termed “impure” (Andreoni, 1989; Batson and Shaw, 1991; Krishna, 2011). The personal benefits derived from “impure altruism” might be the “warm glow effect” (Andreoni, 1990), the “joy of giving” (Bénabou and Tirole, 2006), moral satisfaction (Kahneman and Knetsch, 1992), a kind self-view (Walster et al., 1973), a self-image of “doing the right thing” (Dawes and Thaler, 1988), making an impact on others’ lives (Duncan, 2004), signaling social status, seeking recognition and appreciation, or acquiring social influence (Wang and Tong, 2015). “Impure” altruism is thus self-focused, while “pure” altruism is essentially other-focused (Barasch et al., 2014).

Relying on the distinction between individualistic and collectivist cultures, we propose that the dominant form of altruism differs across these cultures. Individualism’s emphasis on the self and on self-enhancement leads to making decisions that are instrumental to one’s own self (Kitayama et al., 1995). This would be true for any behavior, including altruistic behavior. That is, individualists behave altruistically to serve their own goals (Kemmelmeier et al., 2006; Luria et al., 2015). This view is reflected in the first quote at the beginning of the Introduction section. By contrast, collectivism emphasizes interdependence and embeddedness with in-group members (Delle Fave et al., 2016). This prompts collectivists to be constantly tuned in to others’ needs and to behave altruistically to benefit or serve others. This view of selfless altruism is reflected in the second quote. Thus, people in individualistic and collectivist cultures hold distinct focal motivations for altruism: for collectivists, altruism is focused on the beneficiary and is therefore considered more “pure” (Barrett, 2015), whereas in individualistic cultures, altruism is focused on the helper and is considered “impure” (Kemmelmeier et al., 2006).

The empathy–altruism hypothesis (Batson et al., 2015) reinforces our argument. This theory suggests that altruism may be driven by feelings of empathy—an emotion that focuses on the other. The cultural psychology literature suggests that other-focused emotions (i.e., emotions possessing other people as the primary referent, which foster interdependence) are more pronounced in collectivist cultures, while ego-focused emotions (i.e., emotions possessing one’s own internal attributes, which foster independence) are more pronounced in individualistic cultures (Markus and Kitayama, 1991). Thus, whereas ego-focused emotions are more frequently and intensely expressed and experienced among individualists, other-focused emotions (such as empathy) are more frequently and intensely expressed and experienced among collectivists. Collectivists’ developed empathetic concerns, therefore, lead them to engage in “pure” altruism, while individualists’ focus on the self inclines them toward “impure” altruistic behavior.

Indeed, research has demonstrated cultural differences in people’s views on helping (Septianto et al., 2021). In India (a relatively collectivist society; Hofstede et al., 2010), people believe that altruism involves prioritizing society’s needs *over one’s own*. In Italy (a relatively individualistic society; Luria et al., 2015), altruism arises out of selfish motivations (Soosai-Nathan et al., 2013). Thus, we hypothesize:

*H1.* People with a collectivist cultural orientation display a more “pure” form of altruism, while people with an individualistic cultural orientation display a more “impure” form of altruism.

### The altruism-happiness link through a cultural lens

Collectivists’ tendency toward pure altruism means that their altruistic behavior is focused on benefiting others (rather than themselves). Furthermore, collectivists are practiced in being responsive to others and thus do so intuitively and unintentionally (Riemer and Shavitt, 2011; Riemer et al., 2014). Because collectivists’ altruistic behavior is frequent (e.g., Espinosa et al., 2022) and unintended, they tend not to devote a great deal of attention to such behavior, which in turn limits the potential of the behavior to impact on the helper’s internal state (Taylor and Fiske, 1978). Therefore, we argue there is a relatively low likelihood that collectivists’ altruistic behavior will enhance the helper’s own happiness. By contrast, individualists’ more impure view of altruism means that their altruistic behavior is focused on benefiting themselves (rather than others). Moreover, individualists are not only focused on enhancing their own selves in general but also ascribe great importance to enhancing their own happiness (Oishi and Diener, 2001; Delle Fave et al., 2016). Enhancing happiness is, thus, a prime motivation for altruist behavior among individualists but less so among collectivists. Individualists’ altruistic behavior is, therefore, more intentional and unusual and thus more salient, and it attracts more attention. This, in turn, enhances the potential impact of altruistic behavior on the helper’s internal state, and particularly on their level of happiness (Taylor and Fiske, 1978). Consequently, we propose altruistic behavior is more likely to enhance personal happiness in individualists than in collectivists. Past research on cultural differences in the frequency and dynamics of altruistic behavior, in various motivations for altruistic behavior (values, norms, and self-view), and in terms of the pursuit of happiness provides the basis for our proposition regarding cultural differences in the effect of helping on happiness, as discussed below.

#### The frequency and dynamics of helping

The frequency with which people engage in altruistic behavior differs across cultures, depending on the strength of the social ties within the culture and on the nation’s wealth (Aknin et al., 2013a; Espinosa et al., 2022). In nations with strong social ties and high personal security (i.e., greater trust in others), people engage more in helping strangers, donating money to charity, and informal volunteering (Smith, 2015). Social ties are known to be stronger in lower socioeconomic classes and in collectivist cultures (Carey and Markus, 2016), and consistently, helping is less frequent in wealthy individualist countries (Levine et al., 2001; Van de Vliert et al., 2004). Cultures also differ in the dynamics of the process by which people engage in helping. Collectivists are more likely to engage in spontaneous helping, while individualists tend to be involved in well-planned helping (Aydinli et al., 2013).

#### The motivations for helping behavior across cultures

Culture distinguishes between societies in terms of values, norms, and self-concepts, all of which may determine people’s motivations to become involved in altruistic behavior. First, cross-cultural differences in the importance of distinct values affect motivation for various sought-after goals (Levontin and Bardi, 2019). Individualistic cultures promote values such as achievement and personal conscience. Collectivist cultures, by contrast, promote values such as group cohesion, social harmony, and conformity (Lu and Gilmour, 2004). Consequently, individualists are motivated to engage in activities that improve self-management skills, match personal interests, and provide rewards, while collectivists are motivated to engage in activities that enhance connections with their social group (Gherghel et al., 2020). These tendencies can also explain cultural differences in the frequency of altruistic behavior, as mentioned above.

Second, cultures differ in the extent to which people are attuned to social norms (e.g., Fischer et al., 2019). Individualistic cultures prioritize personal liberties. They subordinate in-group goals to the individual’s goals. Collectivist cultures, however, emphasize social norms and subordinate people’s personal goals to conform to social expectations (Triandis, 1995; Kawamura and Kusumi, 2020). Collectivist societies frequently define and dictate “proper” behavior, relying on a threat/reward system, such as social coercion, to prompt people to comply with the norm (Ahuvia, 2002; Kawamura and Kusumi, 2020). Indeed, Finkelstein (2011) found that whereas individualists engage in helping their workplace to enhance its marketplace value, thereby promoting their own success, collectivists do so because they value group loyalty and adhere to group norms: they feel committed to their colleagues, and they help others as part of their social obligation. Moreover, collectivists not only tend to behave in line with the norm; they are also practiced in doing so, doing it intuitively and automatically (Riemer and Shavitt, 2011; Riemer et al., 2014). This means that the fulfillment of social obligations is not directed toward changing their own internal state (i.e., their happiness). Thus, when fulfilling social obligations, collectivists tend not to be attentive to changes in their internal state, which limits the potential of such behaviors to impact on their internal state (Taylor and Fiske, 1978).

Third, cultures differ in the extent to which people’s definitions of self include (or do not include) other people. Individualists define themselves in terms of their uniqueness and separateness from others and act in line with their internal states (i.e., dispositions and emotions). By contrast, collectivists define themselves in terms of their interdependence with their in-group, relatedness, and unity with others (Markus and Kitayama, 1991; Singelis, 1994; Singelis et al., 1995; Fiske et al., 1998). Collectivists are highly motivated to adjust themselves to the social context (Markus and Kitayama, 1991; Riemer et al., 2014) because their self-concept is bounded by it (Markus and Kitayama, 1991; Fiske et al., 1998; Brewer and Chen, 2007), and they do so intuitively (Riemer et al., 2014). Consequently, to help others or act toward fulfilling somebody else’s goals, individualists must have special (and perhaps internal) motivation, making helping more unusual and salient for individualists, and in turn highly likely to influence their internal state (Taylor and Fiske, 1978). By contrast, collectivists are embedded with others, and thus acting toward fulfilling others’ goals is not unique or unusual behavior, and does not attract special attention from the helper’s side (Gómez et al., 2000). Helping, therefore, is less likely to change collectivists’ internal state (it will not enhance their personal happiness).

#### The pursuit of happiness

Lastly, cultural differences in emotions (Mesquita, 2001), particularly those relating to happiness, may also reflect variations in the motivation to help. One’s own happiness, in itself, is an important goal in individualistic cultures, which promotes self-focus, but not in collectivist cultures, which promote an other-focus (Tsai, 2001; Rego and Cunha, 2009). In cultures that glorify happiness, people’s behavioral choices focus on seeking opportunities to enhance their happiness (Lu and Gilmour, 2004). Consequently, behaviors in general, and helping in particular, are likely to result in the helper’s happiness (Aknin and Whillans, 2020). Thus, it is expected that individualists’ helping, which is more likely to be motivated by the enhancement of personal happiness, will ultimately result in happiness. By contrast, collectivists’ helping, which is more motivated by the enhancement of other people’s wellbeing, is less likely to result in the helper’s personal happiness.

In summary, the research reviewed here suggests differences between individualists and collectivists in the frequency, dynamics, and motivations of helping behaviors, as well as in the pursuit of happiness. According to this body of research, compared to individualists, collectivists engage in helping more often, do so more intuitively, and focus more on the recipient than on themselves, thus ignoring their own emotional benefit. Hence, we hypothesize:

*H2*. Helping is more likely to increase happiness among individualists than among collectivists.

## Current studies

We conducted four studies. Study 1 tested Hypothesis 1 regarding cultural differences in the display of pure and impure altruism. Studies 2 and 3 employed lab experiments to examine the moderating role of cultural orientation in the effect of helping on happiness. Study 4 used a data set from the World Values Survey and reinforced the moderating role of culture in the altruism–happiness link.

Notably, individualistic and collectivist cultures promote the development of enduring cultural orientations in people, leading to differences in their orientations. Individualistic and collectivist orientations are more salient in Western and non-Western cultural contexts, respectively (Markus and Kitayama, 1991; Schwartz, 1992; Triandis, 1995). Further, people with varying degrees of individualistic and collectivist orientation can be found in all geographic areas, although they are not distributed evenly within cultures (Wang, 2008; Cross et al., 2011). Researchers have used various operationalizations to examine cross-cultural differences, including comparing Western (e.g., Americans) with non-Western (e.g., Asian; Ji et al., 2000) cultures or measuring cultural orientation at the individual level within a specific society (Riemer et al., 2014). Measuring cultural orientation at the individual level utilizes within-country variations in cultural orientation to understand the role of the individualism–collectivism value in various psychological phenomena. However, because individualistic and collectivist orientations are rooted in cultural practices (Markus and Kitayama, 2010), even when measured at the individual level, they are considered cultural factors rather than merely individual tendencies (Singelis, 1994; Na et al., 2020). Along these lines, the first three studies operationalized culture using an established individual measure of cultural orientation (Triandis and Gelfand, 1998), whereas the last study operationalized culture at the country level (Bang et al., 2021), relying on cultural orientation measurement across countries by Hofstede (2011). All measures, manipulations, and methods of determining the sample size are disclosed.

## Study 1: cultural differences in the notion of altruism

This study examined cultural differences in the notion of altruism. It tested Hypothesis 1, suggesting that compared to collectivists, individualists tend to display a less ‘pure’ form of altruism.

### Methods

#### Participants

A total of 299 Israeli undergraduate students (76.1% female; *M*_age_ = 24.64 years, *SD* = 2.44) participated in this study. They received extra credit points for their course grades. The sample size was determined *a priori* using G*Power 3.1.9.7 (Faul et al., 2007) for correlation analysis to allow detection of a small-to-medium effect (|*ρ*| = 0.15) with alpha at 0.05 and power of 0.80. This calculation detected a required sample size of 270 participants; thus, we aimed to recruit approximately 300 participants.

#### Cultural orientation

Participants’ cultural orientation was determined using the Triandis and Gelfand (1998) scale. The scale contains 16 items: 8 items measure individualism and 8 items measure collectivism. The participants rated their dis/agreement with each statement on a 7-point scale (1 = strongly disagree, 7 = strongly agree; α_individualism_ = 0.80; α_collectivism_ = 0.76). For the analysis, we created an index by subtracting the respondent’s mean score on the items indicating collectivism from those indicating individualism (Riemer and Shavitt, 2011). This index specifies the participant’s orientation on a collectivism–individualism continuum: the higher (lower) the value of the index, the more individualistic (collectivist) the respondent’s orientation is.

#### Altruistic disposition

To examine variations in the display of various facets of altruism, participants completed the following scales, measuring four factors: (1) cynical giving (Furnham, 1995); (2) altruistic giving (Furnham, 1995); (3) altruistic personality (Rushton et al., 1981); and (4) helping attitude (Nickell, 1998).

Cynical giving refers to people’s ulterior helping motivation (Furnham, 1995). People who possess cynical views of altruism believe that people engage in altruistic behavior due to some ulterior motivation, such as calming their guilty consciences (McReynolds, 2013). Such ulterior motives are attributable to selfish incentives to do good (Berman and Silver, 2022). Cynical giving beliefs were measured using three items (e.g., “For many, charity donation is simply a tax dodge”; measured on a 5-point scale: 1 = strongly disagree, 5 = strongly agree; Furnham, 1995). The overall score was determined by the average score across the items. Higher (lower) scores on the cynical giving scale represent a more (less) cynical view of altruism. Thus, a higher score would suggest a display of relatively impure altruism.

Altruistic giving refers to people’s belief that those who donate to charity are *genuinely* altruistic (Furnham, 1995). It was measured using two 5-point items (e.g., “People who give to charity and work for charity are genuinely altruistic”). Higher overall scores (determined by the average across the two items) represent a greater belief in genuine altruism and thus would be considered a display of pure altruism. (Both factors—cynical giving and altruistic giving—are part of a scale that measures attitudes toward charitable giving (Furnham, 1995), which includes five factors. The three other factors on this scale—inefficiency of charitable giving, efficiency of charitable giving, and purpose of charity—are irrelevant for our purpose and were therefore not included in our study).

Altruistic personality refers to a person’s inherent stable trait or altruism; thus, knowing that one possesses this trait would predict that one would behave altruistically in a diverse range of situations (Rushton et al., 1981). It was measured using the Self-Report Altruism (SRA) scale, which gauges the frequency of one’s altruistic behavior (e.g., “I have given directions to a stranger”). Respondents rated the frequency with which they engaged in such behaviors on a 5-point scale (1 = never, 5 = very often). We used 14 out of the original 20 items, eliminating those that were irrelevant to the present time or to the participants’ cultural context (e.g., “I have bought ‘charity’ holiday cards deliberately because I knew it was a good cause”). The elimination process was performed using the Delphi technique, relying on a team of experts—in our case, six independent judges—in two rounds and based on consensus (Dalkey and Helmer, 1963). Participants’ scores on this factor were determined by the average score across items, where higher (lower) scores represented a greater (lower) tendency toward altruistic behavior. We posited that because those who score high on this factor are considered altruist regardless of the situation, they are likely to be considered as displaying more pure altruism.

Helping attitude refers to beliefs, feelings, and behaviors related to helping people (Nickell, 1998). It was measured using the Helping Attitude Scale (HAS), a multidimensional scale that includes 20 items examining beliefs, feelings, and behaviors associated with helping (e.g., “Charity is an intelligent way of distributing money”), rated on a 5-point scale (1 = strongly disagree, 5 = strongly agree). The scores for all items were summed up to form an overall score, ranging from 20 to 100, with 60 being a neutral score; higher (lower) scores represent a more (less) favorable attitude toward helping. Given that this scale measures various facets of disposition toward helping, it is reasonable to assume that high scores represent a constantly more favorable attitude toward helping, and thus display more pure altruism.

### Results

Correlational analyses were used to examine the relationship between participants’ cultural orientation index scores (collectivism–individualism; higher score representing more individualistic orientation) and their scores on the four altruistic disposition factors. Results indicated *negative* relationships between the cultural orientation and scores of altruistic giving [*r*(298) = −0.33, *p* < 0.001, 95% CI [−0.43, −0.23]], altruistic personality [*r*(298) = −0.18, *p* = 0.002, 95% CI [−0.28, −0.06]], and helping attitude [*r*(298) = −0.39, *p* < 0.001, 95% CI [−0.49, −0.29]], and a *positive* relationship between the cultural orientation index and cynical giving [*r*(298) = 0.28, *p* < 0.001, 95% CI [0.17, 0.38]]. This suggests that the more individualistic participants are, the less likely they are to display dispositions of altruistic giving, altruistic personality, and helping attitude, and the more likely they are to be cynical about giving (see Table 1). Thus, supporting Hypothesis 1, a more collectivist orientation is positively associated with tendencies reflecting “pure” altruism, while a more individualistic orientation is positively associated with a cynical view about helping reflecting more “impure” altruism.

**Table 1:** Mean, standard deviation, and Pearson correlation matrix for Study 1 (*n* = 299).

	Mean	*SD*	Cultural orientation	Cynical giving	Altruistic giving	Altruistic personality	Helping attitudes
Cultural orientation	−1.06	1	1				
Cynical giving	2.82	0.70	0.28**	1			
Altruistic giving	3.89	0.65	−0.33**	−0.13*	1		
Altruistic personality	3.13	0.49	−0.18**	0.09	0.19**	1	
Helping attitudes	4.02	0.41	−0.39**	−0.15**	0.44**	0.38**	1

**Correlation is significant at the 0.01 level (2-tailed).

*Correlation is significant at the 0.05 level (2-tailed).

## Study 2

This study aimed to replicate prior studies concerning the effect of helping on happiness while extending to uncover the moderating role of culture in this effect. We used a similar helping manipulation and happiness measure used by Aknin et al. (2013a), and added a measure of cultural orientation as the operationalization of culture.

### Methods

#### Design and participants

This study used a between-subjects experimental design in which the participants were randomly assigned to one of two giving behavior conditions: to oneself or to others. Cultural orientation, the moderating factor in this study, was measured. In total, 229 Israeli students were recruited from the same pool as in Study 1, but none of the participants in the current study participated in Study 1. We excluded 58 participants who failed to follow the instructions. Our final sample size consisted of 171 participants (70.2% female; *M*_age_ = 24.58 years, *SD* = 2.55). Participants performed the giving behavior manipulation and then completed the happiness scale and the cultural orientation measure, all described below. A *post hoc* analysis conducted using G*Power 3.1.9.7 (Faul et al., 2007) suggested that we had approximately 80% power to detect an effect size of *f*^2^ = 0.07.

#### Giving behavior

In line with previous research (e.g., Dunn et al., 2008) suggesting that spending money on others is an expression of giving behavior, we utilized a known manipulation of spending, asking participants to recall and describe a recent event in which they spent money either on themselves or on others (Strack et al., 1985; Aknin et al., 2013a; Bastos, 2020). This manipulation was designed to elicit vivid reminiscence for 120 s. Participants were instructed: “Try to recall a recent event in which you spent money on yourself (on others). Describe the experience in as much detail as possible.”

#### Happiness

Similar to research by Aknin et al. (2013b), happiness was measured using the 4-item Subjective Happiness Scale (Lyubomirsky and Lepper, 1999; *α* = 0.88). For each item, the participants completed a sentence fragment by choosing one of seven options according to what they viewed as most appropriate for them. This scale is widely used and has proven to be reliable for measuring happiness and correlated with other measures of subjective wellbeing and happiness (Lyubomirsky and Lepper, 1999; Shahen et al., 2019). The overall score was determined by the mean across the four items, with higher (lower) scores representing greater (lesser) happiness.

#### Cultural orientation

Similar to Study 1, participants completed the cultural-orientation scale (Triandis and Gelfand, 1998), and we used an index score indicating participants’ score on the collectivism–individualism continuum (Riemer and Shavitt, 2011), in which the higher (lower) the value of the index, the more individualistic (collectivist) the respondent’s orientation is.

### Results

The happiness score was significantly lower for the participants in the self-spending condition compared to those in the spending on others condition [*M*_self_ = 4.83, *SD* = 1.23*, M*_others_ = 5.19, *SD* = 0.98, *t*(158.4) = −2.08, *p* = 0.039, *d* = −0.32, 95% CI [−0.62, −0.02]]. This finding replicates the main effect found in past studies, showing that spending on others leads to greater happiness than spending on oneself (e.g., Aknin et al., 2018).

**Figure 1:**
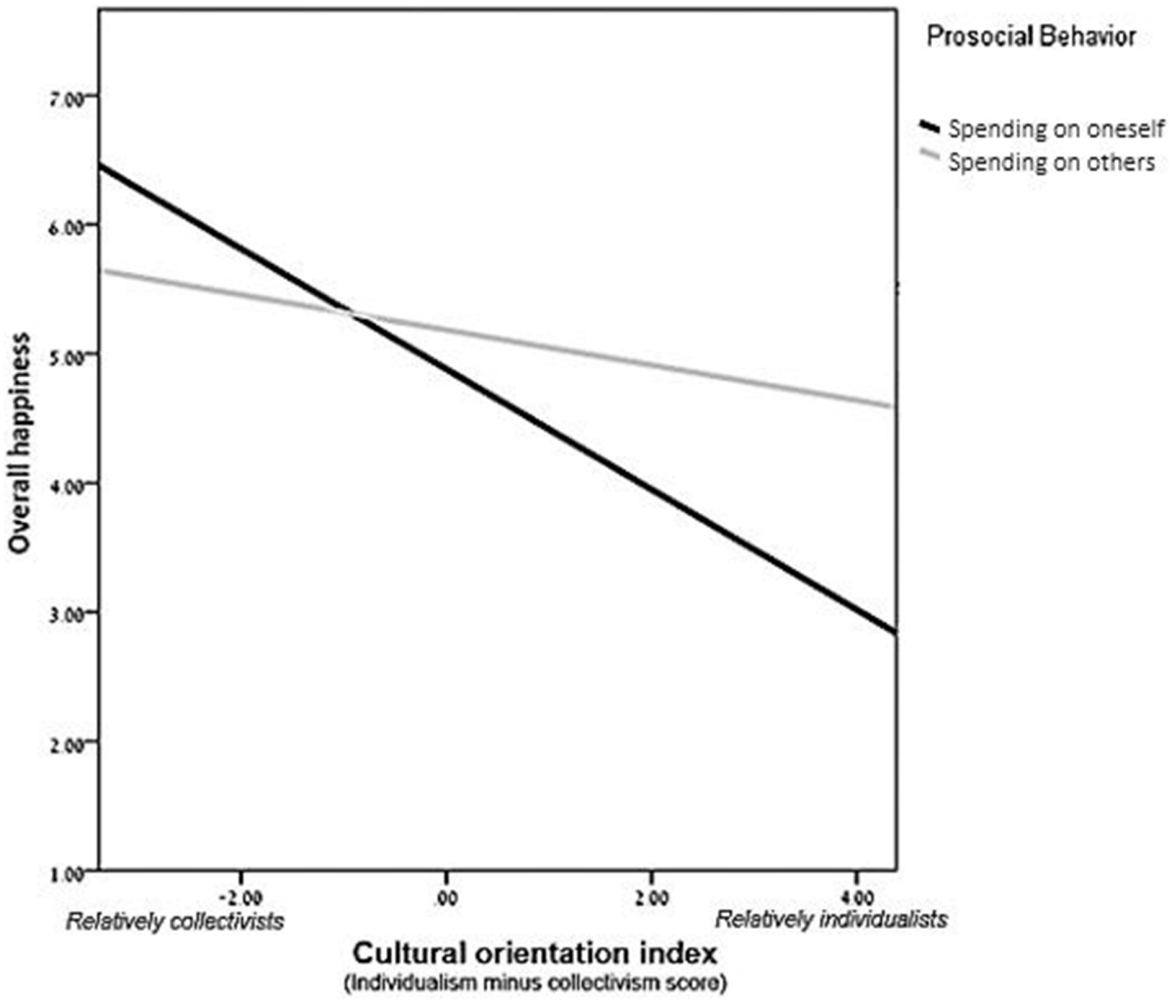
Scores on Subjective Happiness Scales as a function of cultural orientation index and prosocial behavior: Study 2. *Higher scores on the cultural orientation index signify a more individualistic (and less collectivist) orientation.

To determine whether cultural orientation moderated the effect of spending type on happiness, we performed a regression analysis on respondents’ happiness with the following independent variables: (i) a cultural orientation index, (ii) a dummy variable for spending type (0 = self; 1 = others), and (iii) the interaction of these variables. The linear regression model was significant [*F*(3, 167) = 8.73, *p* < 0.001, *f*^2^ = 0.16, *R*^2^ = 0.14, *R*^2^_adjusted_ = 0.12], and the interaction between cultural orientation and spending type was significant (*β* = 0.196, *p* = 0.041). To further explore the interaction, we performed spotlight analyses (Aiken and West, 1991; Fitzsimons, 2008), spotlighting the results of participants with above-zero scores on the cultural orientation index (a relatively individualistic orientation) versus those with below-zero scores (a relatively collectivist orientation). The analyses revealed that for those with a relatively individualistic orientation, spending on others led to increased happiness compared to spending on oneself (*β* = 0.28, *p* = 0.009). In contrast, for those with a relatively collectivist orientation, spending type did not play a role in happiness (*β* = −0.028, *p* = 0.79; Figure 1). These results point to a cultural difference in the effect of altruist behavior on happiness and support Hypothesis 2.

## Study 3

This study tested the same effect examined in Study 2 but with a different form of help: a kind gesture, that is, preparing tea. This method was adapted from the “Starbucks study” (Norton et al., 2010; Aknin et al., 2011), in which participants received Starbucks gift cards to buy coffee either for themselves or for a friend. In our study, coffee was replaced with green tea, and the procedure was slightly modified, as described below.

### Methods

#### Design and participants

Similar to Study 2, this study used a between-subjects experimental design, in which participants were randomly assigned to one of two giving behavior conditions: to oneself or to others, and cultural orientation, the moderating factor in this study, was measured. The participants were 251 Israeli students (80.1% female; *M*_age_ = 24.08 years, *SD* = 1.48); they belonged to the same pool of the previous studies but did not participate in those studies. They were randomly assigned to one of the giving behavior conditions. They then completed the happiness scale and the cultural orientation measure used in Study 2. An *a priori* power analysis using G*Power 3.1.9.7 (Faul et al., 2007) indicated that the required sample size was 160 to detect a small-to-medium effect (*f*^2^ = 0.07), with α = 0.05 and power of 0.80. Given that this was a two-part study, we assumed approximately 30–40% attrition and therefore aimed to recruit 260 participants. Surprisingly, 251 participants performed this experiment in its entirety (i.e., both parts).

#### Manipulating giving behavior

The participants arrived at our behavioral lab during the morning hours. They read a passage describing the value of green tea and were instructed to prepare tea either for themselves or for another person (depending on the giving behavior condition) at a later time on the same day (before 6 p.m.). Upon leaving the lab, the participants received an envelope containing a green tea bag, printed information identical to the passage they had read, and a reminder of whom they should prepare it.

#### Measuring happiness

In the evening of the same day, the participants received an e-mail containing a link to a questionnaire. In this questionnaire, they were asked to think about the preparation of the tea earlier and describe in detail their experiences of making it. They then completed the Subjective Happiness Scale used in Study 2 (Lyubomirsky and Lepper, 1999; *α* = 0.83).

### Results

**Figure 2:**
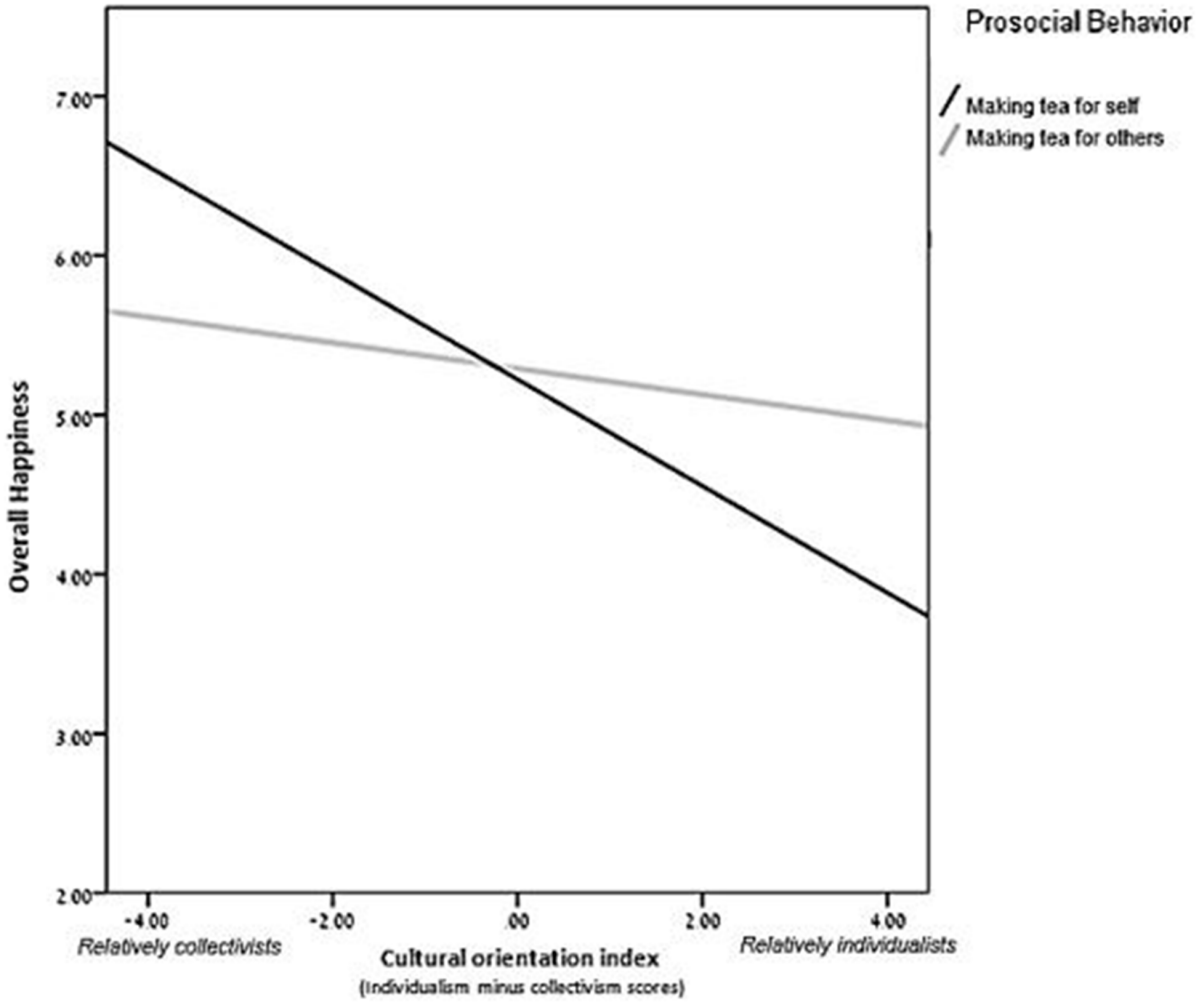
Scores on Subjective Happiness Scales as a function of cultural orientation index and prosocial behavior: Study 3. *Higher scores on the cultural orientation index signify a more individualistic (and less collectivist) orientation.

The main effect of the giving condition on happiness was insignificant [*M*_self_ = 5.23, *SD* = 1.04*, M*_others_ = 5.29, *SD* = 0.91, *t*(249) *=* 0.45*, p* = 0.652, 95% CI [−0.19, 0.299]]. A regression analysis was performed on respondents’ happiness scores with independent variables: (i) the cultural orientation index, (ii) a dummy variable for giving behavior (0 = self; 1 = others), and (iii) their interaction. The results showed a significant interaction between cultural orientation and giving behavior [*F*(3, 247) = 8.806, *p* < 0.001, *f*^2^ = 0.11, *R*^2^ = 0.097, *R*^2^_adjusted_ = 0.086, *β* =0.208, *p* = 0.012]. To explore the interaction, we performed spotlight analyses spotlighting the results of participants with above-zero scores on the cultural orientation index (individualists) versus those with below-zero scores (collectivists). The analyses revealed that for individualists, giving others led to increased happiness compared to giving oneself (*β* = 0.19, *p* = 0.028). For collectivists, the giving condition did not affect happiness (*β* = −0.12, *p* = 0.163; Figure 2). These results reinforce the role of culture in the effect of altruist behavior on happiness (Hypothesis 2).

## Study 4

Study 4 aimed to strengthen our findings by operationalizing cultural orientation on a country level relying on cultural orientation measurement across countries (Hofstede, 2011) rather than on an individual level. This study utilized the World Values Survey (WVS, Wave 7: 2017–2021)—a cross-national survey measuring attitudes, beliefs, and behavior patterns of diverse populations. The survey has been administered every 5 years since 1981 using newly selected cross-sectional samples. For our purpose, we used two questions: a question about whether the respondent is active in donating to charities (a proxy for altruist behavior tendency) and a question about whether the respondent feels generally happy (an indication for happiness). The association between these two measures serves as an indication of the link between helping and happiness, and it was examined across cultures using countries’ cultural orientation scores (COS) by Hofstede (2011).

### Methods

#### Participants

The WVS database included 49 countries and 70,867 participants (51.7% female; *M*_age_ = 42.4 years, *SD* = 16.39). Country of residence was used as an indication of culture according to the individualism–collectivism continuum index (Hofstede Culture Compass™; Hofstede, 2011). Eight countries that did not have a score on that index were excluded, leaving a sample of 61,705 participants (51.8% female; *M*_age_ = 42.6 years, *SD* = 16.45) from 41 countries. The final sample size, after excluding missing values and additional participants based on the criteria described below, consisted of 42,288 participants (52.1% female; *M*_age_ = 43.3 years, *SD* = 16.5). Since we used an existing database with a set number of participants, a *post hoc* analysis was conducted with G*Power 3.1.9.7 (Faul et al., 2007), suggesting maximum (100%) power for any effect size for this large sample size.

#### Measures

The participants’ altruistic behavior tendency was determined based on their responses to the question pertaining to whether they donated to a group or social campaign. The response options were as follows: would never do, might do, and have done. We eliminated participants who responded “might do,” as it indicates intended future behavior that may or may not occur. In addition, the motivations for choosing this response might be different (e.g., social pressure or positive self-image; Shephard, 2003; Brenner and DeLamater, 2016), not necessarily reflecting authentic intentions, possibly distorting the results. This step resulted in the exclusion of 17,404 participants. The two remaining response options represented the two extremes of altruistic behavior, enabling us to classify respondents into two clear-cut categories: those who engage in altruistic behavior and those who do not. We also eliminated 1,918 participants who did not answer this question (missing data).

Happiness was determined based on a direct question: “Taking all things together, would you say you are: very happy, quite happy, not very happy, not at all happy.” Each option was coded on a four-point scale (1 = not at all happy, 4 = very happy). There was also a “do not know” option, which no participant selected, and six respondents did not answer the question and therefore were eliminated from the analysis.

### Results

Taking into account the nested structure of the data, we applied the multilevel modeling approach (MLM) for the analysis. This analysis examines the relations between helping behavior and cultural orientation on happiness at both the individual- and the country-level, as well as the interactions between helping and cultural orientation at both levels. The detailed analysis is described below (see also Table 2). First, we ran an intercept only model to determine whether the data is suitable for MLM. This null model partitions the variance in the dependent variable (happiness) into its individual-level and country-level components. Results show that individuals’ variability in happiness (*σ*^2^ = 0.46) accounts for 89.85% of the overall variance, and the between-countries variance is significantly non-zero (*τ* = 0.052, Wald *Z* = 4.917, *p* < 0.001), suggesting that substantial amount of the variance lies at the between countries level (ICC = 0.10). According to Heck et al. (2013), a multilevel model should be applied when ICC is larger than 0.05, so we continued to the next levels. To ease coefficient interpretation, prior to analyses, all individual-level predictors were group mean centered and between-countries predictors were grand mean centered (Enders and Tofighi, 2007).

**Table 2:** HLM analysis: effects of helping on happiness across cultures.

	Null model	Model 1	Model 2	Model 3	Model 3a effect of individualists	Model 3b effect of collectivists
Individual level						
Intercept	3.15^***^	3.15^***^	3.14^***^	3.10^***^	2.96^***^	3.24^***^
Helping		0.032^*^	0.028	0.028		
Country level						
Culture (COS)			−0.002	−0.007^*^		
Helping (aggregated)			0.166	0.228	0.664^*^	−0.208
Helping × COS				2.235^e-5^		
Helping (aggregated) × COS				0.021^*^		
Variance components						
𝜎^2^	0.46	0.46	0.46	0.46		
τ	0.05	0.05	0.04	0.03		
*R*^2^ (within)		0.00				
*R*^2^ (between)			0.23	0.34		

^*^p < 0.05, ^***^p < 0.001.

The level 1 analysis was conducted with the individual-level helping tendency as the predictor. This analysis yielded an intercept of 3.149 (*SE* = 0.0*3*4, *t* = 93.395, *p* < 0.001), and a significant association between helping tendency and happiness (*B* = 0.032, *SE* = 0.016, *t* = 2.023, *p* = 0.043). For the level 2 analysis we added the country-level variables, that is: the cultural orientation score (COS) and the aggregated country-level helping tendency reflecting the average score of each country in helping tendency. This analysis revealed an intercept’s estimate of 3.138 (*SE* = 0.032, *t* = 97.249, *p* < 0.001); the individual-level helping tendency is a marginally significant predictor (*B* = 0.028, *SE* = 0.016, *t* = 1.747, *p* = 0.081); COS is an insignificant predictor (*p* = 0.202); and the country-level helping tendency is an insignificant predictor (*p* = 0.242). Next, we added to the model the interaction between COS and helping tendency at the individual level, as well as the interaction between COS and helping tendency at the country level. This analysis revealed an intercept’s estimate of 3.099 (*SE* = 0.035, *t* = 88.692, *p* < 0.001); the interaction between COS and helping at the individual level is insignificant (*B* = 2.2359^e-05^, *SE* = 0.001, *t* = 0.028, *p* = 0.978); while the interaction between COS and helping at the country level is significant (*B* = 021, *SE* = 0.009, *t* = 2.179, *p* = 0.030). To probe the significant interaction, we estimated the helping-happiness association at high (M + 1SD) and low (M − 1SD) levels of COS.

This simple slopes analysis reveals that for individualists (i.e., high COS), helping tendency is significantly and positively linked to happiness (*B* = 0.664, *SE* = 0.269, *t* = 2.466, *p* = 0.014), but for collectivists (i.e., low COS) this link is insignificant (*B* = −0.208, *SE* = 0.216, *t* = −0.962, *p* = 0.336). Interestingly, the results of this study provide support to our predictions only at the country level but not at the individual level. This discrepancy between the country- and individual-level results might have to do with ecological fallacy, suggesting that group-level and individual-level analyses do not necessarily provide similar results (Bond, 2002; see also Smith, 2004; Shavitt et al., 2006; Torelli and Shavitt, 2010), which deserves further examination, as will be discussed later.

## General discussion

### Summary and discussion of results

We propose that there are cultural differences in the notion of altruism, such that a collectivist cultural orientation is associated with tendencies reflecting more “pure” altruism, while an individualistic cultural orientation is associated with more “impure” altruism. These different perspectives on altruism are the basis for the distinct effects of altruistic behavior on happiness across cultures: the helping-happiness link holds for individualists but not for collectivists, thus is not a universal phenomenon.

Four studies support our propositions. The first study provides evidence that when one’s cultural orientation is more individualistic (collectivist), one is more likely to display dispositions associated with impure (pure) altruism. Namely, an individualistic (collectivist) cultural orientation was shown to be positively (negatively) associated with a cynical view of altruism and negatively (positively) associated with altruistic giving, altruistic personality, and helping attitude. All correlation coefficients between these various factors and cultural orientations were significant and in the predicted direction. In addition, the correlations between these factors support the premise that all of them are linked to related concepts (display of pure/impure altruism). Yet, it is noteworthy that in one case—the correlation between cynical giving and altruistic personality—the Pearson coefficient was insignificant, suggesting that these factors are not necessarily linked to each other. This might be because the two factors refer to more distant concepts: whereas cynical giving refers to one’s beliefs about altruism in a more general sense, altruistic personality refers to one’s own behavioral tendencies. Although attitude theory would predict an association between the two constructs, research suggests that in a more collectivist cultural orientation, this association may not necessarily hold (Riemer et al., 2014), which may weaken the correlation.

Studies 2–4 use different methodologies, consistently showing that the link between helping and happiness is stronger for those with a more individualistic cultural orientation and weaker for those with a more collectivist cultural orientation. It is noteworthy that in contrast to the results of Study 2 and of past studies (e.g., Aknin et al., 2013a), Study 3 revealed that the main effect of giving behavior on happiness was insignificant. Yet, in both Studies 2 and 3, the results consistently demonstrated that the effect of giving behavior on happiness was significant only for participants with a more individualistic cultural orientation but not for those with a more collectivist one. The fact that in Study 2 the main effect (considering all participants) was significant while being insignificant in Study 3 seems to stem from the nature of the task, which may have determined the strength of the effect. That is, the spending-money manipulation used in Study 2 may have been stronger than the making-tea manipulation used in Study 3, leading to a stronger effect on individualists and thus to a significant main effect, considering all participants.

Interestingly, although Studies 2–4 support our prediction regarding the cultural differences in the link between giving condition (to other vs. to oneself) and happiness, Studies 2 and 3 demonstrate an additional trend. In both studies, individualists and collectivists were equally happy under conditions of giving to others, but individualists were less happy than collectivists under conditions of giving to oneself. This might be because both studies were conducted in Israel, where the general orientation is more collectivist (i.e., less individualistic; Hofstede, 2011), thus the cultural orientation of the individualists in our sample diverged from that of the general population. This misalignment (between the individual-level and the country-level cultural orientations) might lead to reduced happiness after engaging in self-care. That is, for people with an individualistic cultural orientation, the collectivist cultural context may reduce the emotional benefit of giving. This effect is worth examining in future research.

Finally, Study 4 demonstrates a discrepancy between results at the individual level and results at the country level. Specifically, while the country-level analysis yielded results consistent with our prediction and with Studies 2 and 3, the individual-level analysis revealed only significant association between helping and happiness but not cultural differences in the helping–happiness link. As discussed earlier, the discrepancy between the country- and individual-level results might be due to ecological fallacy (Bond, 2002; see also Smith, 2004; Shavitt et al., 2006; Torelli and Shavitt, 2010). This issue calls for more research on individual- versus group-level analyses of cultural phenomena, and particularly on trends of altruistic behavior at the country level.

### Implications

This research carries theoretical and practical implications. First, it sheds light on the role of culture in the notion of altruism and proposes that culture is a key factor in helping behavior. The different concepts of altruism embedded within different cultures suggest distinct motivations—and therefore different consequences—for altruistic behavior.

Our work contributes to the research on people’s motivations. It points to two types of central motivation for altruistic behavior, which might be termed personal and social motivations; each of them is dominant in different cultures. Our findings specifically imply that people in individualistic (vs. collectivist) cultures tend more to seek happiness, and are therefore more motivated to act to achieve it through helping others. This trend is generalizable more broadly to motivation for personal benefits beyond happiness, as well as to behavioral tendencies beyond helping. Indeed, a study by Hornsey et al. (2018) showed that people in collectivist cultures strive more to gain social benefits than people in individualistic cultures, but aspire less to gain personal benefits. Furthermore, both types of motivation (personal and social) can drive similar behaviors, yet lead to distinct consequences of a specific behavior. That is, differences in motivational foci across cultures may result either in cross-cultural variability in the tendency to engage in a certain behavior or in different consequences of the same behavior. Yet in the context of helping, it may very well be that helping behavior that combines social interaction does, in fact, enhance happiness among collectivists. This direction merits further research.

Our findings also imply cultural differences in the sense of happiness. Culture plays a central role in how feelings and emotions are processed, experienced, and expressed (Uchida and Oishi, 2016; Mesquita, 2022). Considering this, the notion of “happiness” may be perceived differently across cultures and is, therefore, pursued in different ways. Differences in the expression of happiness might stem from distinct perceptions of what happiness means (Fang et al., 2019). Our findings reinforce this view, suggesting that culture not only affects the level of happiness in a specific context, but also determines the factors creating happiness or the meaning of happiness. More research should be devoted to this topic.

In addition to its theoretical contribution to research on happiness, the present research offers practical implications for people seeking to enhance their subjective wellbeing. When seeking ways to enhance happiness, people often think about engaging in volunteering activities. Our findings suggest that such behavior would increase happiness in people with an individualistic orientation but not in people with a collectivist orientation. Thus, collectivists should either choose other activities or attempt to change perspective on their altruistic behavior.

This research may also offer insights for charity funds and non-profit organizations as they develop their fundraising campaigns. Our findings may assist in developing accurate culture-specific advertising appeals to promote donations and helping behaviors. In individualistic cultures, emphasizing the emotional benefits of helping might be effective (e.g., “help others, be happy”). By contrast, in collectivist cultures, other appeals may be needed (e.g., “doing the right thing,” “fulfilling your obligation”).

### Further research

Future research should be devoted to examining additional boundary conditions for the effect of helping on happiness across cultures. For example, research should explore the correspondence between the goals of helping and social values, given that the fit between one’s personal values and the activity of a charitable organization has been shown to increase the likelihood of donation (Bennett, 2003). The emotional aspects of such fit merit examination. It is worth exploring whether the extent to which a helping act fits with one’s values influences the level of happiness (or other emotional consequences). Research should also investigate the differences in happiness when helping members of the in-group as opposed to members of the out-group among both individualists and collectivists. Collectivists tend to be inherently committed to in-group members and thus tend to help people in close relationships (Ogihara and Uchida, 2014). Therefore, collectivists’ altruistic behavior is significantly affected by whether the receivers are viewed as in-group or out-group members (Leung and Bond, 1984; Hui et al., 1991). Individualists, by contrast, have weak social ties with both in-group and out-group members (Kasser and Ryan, 2001; Ahuvia, 2002) and demonstrate equivalent disposition toward helping people in both groups (Duclos and Barasch, 2014). Future research should consider whether helping in-group and out-group members yields different emotional consequences for collectivists and individualists.

Notably, measuring happiness in our studies relied on scales developed and validated through research based mostly on a Western-individualistic perspective (Lu et al., 2001). Yet, because the very notion of happiness might differ across cultures (Lu and Gilmour, 2004; Uchida and Kitayama, 2009; Delle Fave et al., 2016; Monroe et al., 2018; Triandis, 2018), future research should aim for alternative scales reflecting these differences. Different cultures may idealize distinct types of expression of happiness (e.g., eudemonic, hedonic, and wellbeing) to various extents (Deci and Ryan, 2008). Future research should, therefore, expand the examination of the effects of altruism and helping on various facets or types of happiness.

In addition, our research focused on one personal consequence of helping behavior: happiness. Helping behavior may lead to other outcomes (Curry et al., 2018), which should be examined across cultures. Future research might, for example, consider experiences of autonomy or self-efficacy resulting from helping, or the role of altruist behavior in the regulation of negative emotions (see Espinosa et al., 2022). Moreover, future research should examine the effect of helping not only on individual-level factors but also on group-level factors, such as harmony, cohesion, and cooperation.

Future research may also look into the underlying mechanism of the effect of helping on happiness. It would be particularly interesting to examine whether the effect is intentional, in the sense that people in individualistic cultures engage in helping with a conscious motivation to enhance their happiness, and whether awareness of this effect might make a difference.

Lastly, the current research relies on Hofstede (2001) individualism–collectivism cultural dimension, which has been supported throughout the years (e.g., Minkov and Kaasa, 2021). Yet, societal changes over the years, particularly following the COVID-19 pandemic, may have shifted cultural orientations in various countries and caused changes in altruistic behavior and happiness (e.g., Rajkumar, 2023). The role of the pandemic in these factors and in the relationships among them merits examination.

## Data availability statement

The raw data supporting the conclusions of this article will be made available by the authors, without undue reservation.

## Ethics statement

The studies involving human participants were reviewed and approved by Ben-Gurion University of the Negev IRB committee, number: HR_2412017. The patients/participants provided their written informed consent to participate in this study.

## Author contributions

The research is part of a doctoral dissertation of MW-S, supervised by HR. MW-S developed the research idea, collected and analyzed the data, and drafted the manuscript. HR contributed to the conception and design of the work, reviewed and revised the manuscript. All authors approved the submitted version.

## Funding

This research was supported by the ISRAEL SCIENCE FOUNDATION (grant No. 1138/22 to HR).

## Conflict of interest

The authors declare that the research was conducted in the absence of any commercial or financial relationships that could be construed as a potential conflict of interest.

## Publisher’s note

All claims expressed in this article are solely those of the authors and do not necessarily represent those of their affiliated organizations, or those of the publisher, the editors and the reviewers. Any product that may be evaluated in this article, or claim that may be made by its manufacturer, is not guaranteed or endorsed by the publisher.
